# Supplementary material for: Shrimp Parvovirus Circular DNA Fragments Arise From Both Endogenous Viral Elements and the Infecting Virus
Source: Front Immunol. 2021 Sep 28;12:729528. doi: 10.3389/fimmu.2021.729528 (PMC8507497; doi:10.3389/fimmu.2021.729528)
Supplement: Supplementary file 1 [file DataSheet_1.pdf]

Supplementary figures

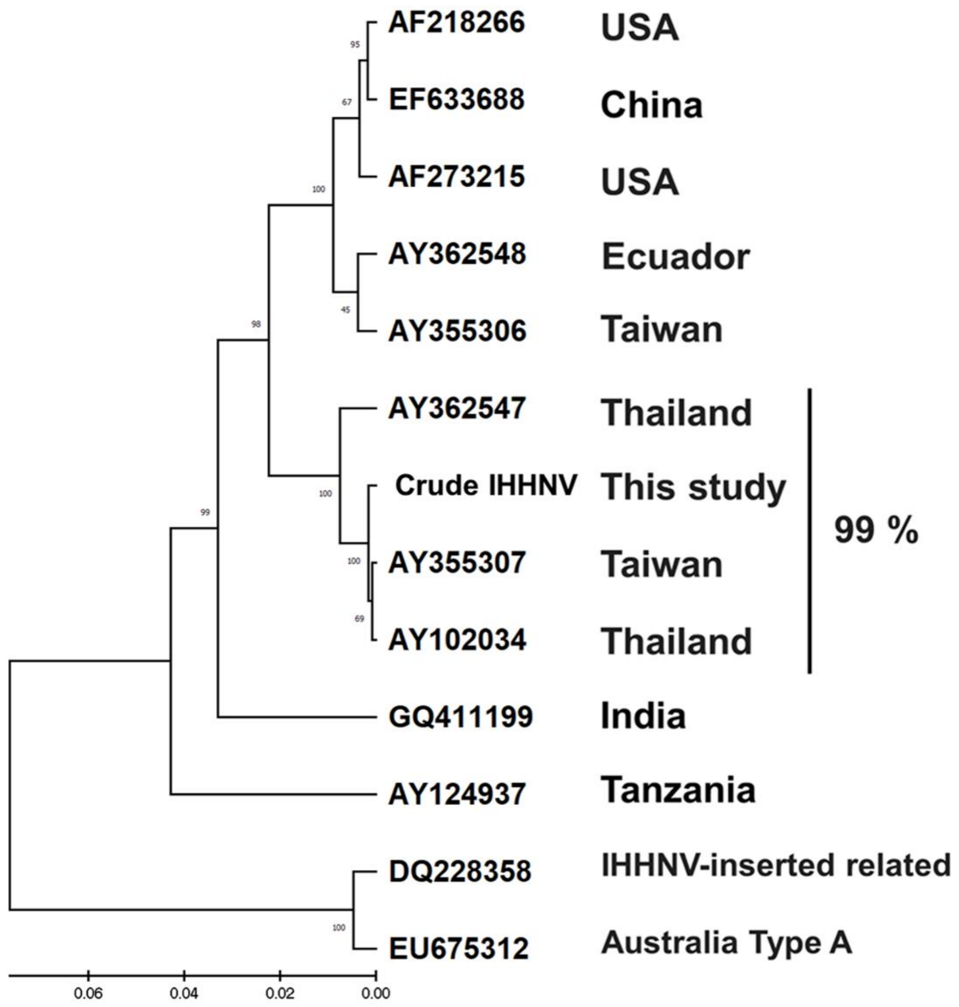

Figure S1. UPGMA cluster tree constructed for comparison of 2814 bases of our nucleic acid sequence (GQ475529) with matching regions of IHHNV sequences at GenBank. The scale bar below the tree indicates the relative difference in percent identity. The vertical bars on the right of the tree indicate the percentage or percentage range of differences in identity among the isolates covered by the bars. Since the GenBank sequences AY355306 and AY355308 from Taiwan were identical in the region compared, only the sequence of AY355306 was used in constructing the tree.

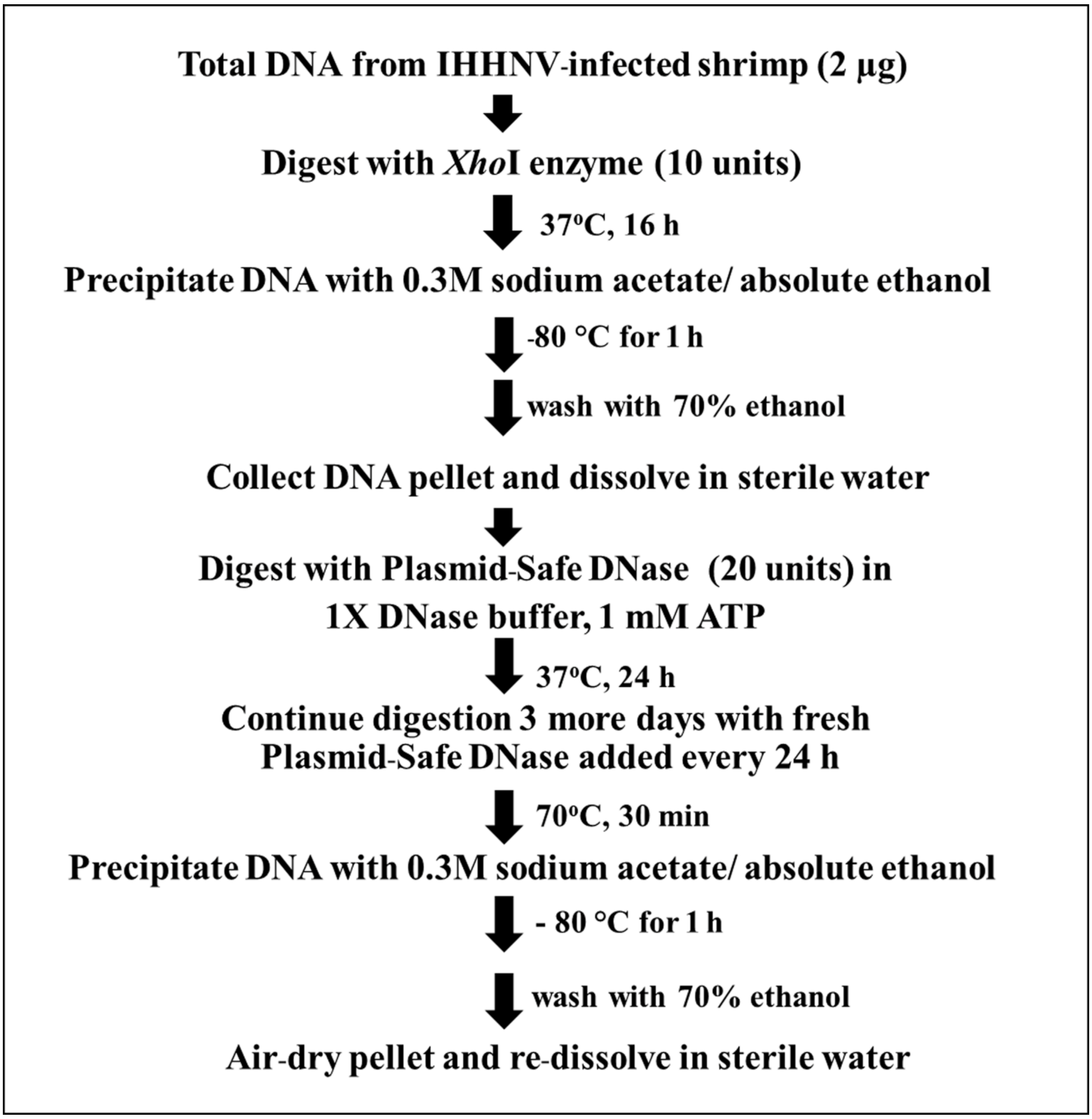

Figure S2. The protocol used to prepare circular DNA from the infected shrimp. Two micrograms of total DNA extract were obtained from IHHNV-infected shrimp by using commercial available DNA extraction kit (Qiagen, Germany). The total DNA was pre-digested with *Xho*I to enhance the digestion efficiency of plasmid-safe DNase in next step. Subsequently, pre-digested DNA was extracted and further incubated with 20 U plasmid-safe DNase (PS-DNase, Lucigen®, Epicentre, UK) and 1mM ATP at 37 °C for 24 h. This digestion step was repeated for 4 days with addition of 20U PS-DNase and ATP every 24h. After that, the reaction was heat inactivated at 70 °C for 30 min and then the remaining putative circular DNA was precipitated by 0.3 M sodium acetate in absolute ethanol. The total yield of circular DNA was dissolved in DNase-RNase free water and then the concentration of DNA was determined by Qubit fluorometer (Invitrogen, USA).

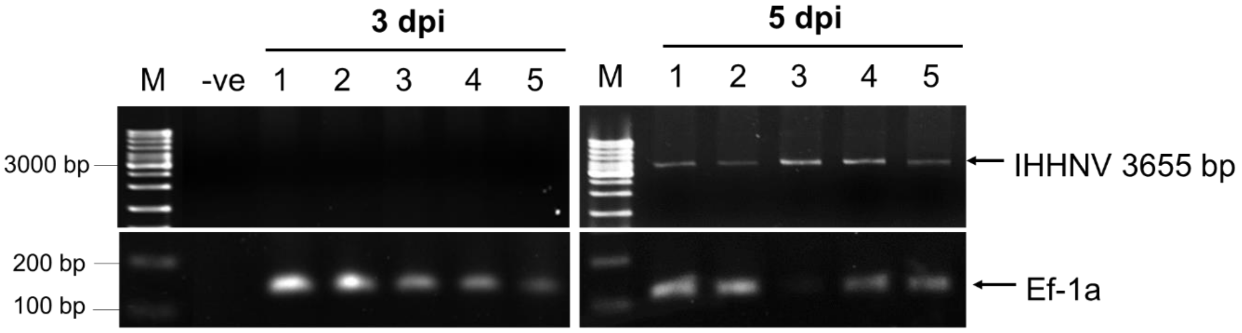

Figure S3. Infectivity testing of the crude IHHNV stock. To test infectivity of the IHHNV stock derived from IHHNV-infected shrimp, 10 naïve *Penaeus vannamei* were injected intramuscularly with 50 µl of the diluted stock (1 x 10^7 copies of IHHNV). At days 3 and 5 post injection, gills from 5 shrimp were collected arbitrarily and their genomic DNA was extracted and subjected to the long-amp PCR analysis method to determine IHHNV genome replication. Five shrimp injected with the stock gave negative test results for IHHNV on day 3 but positive test results on day 5, indicating IHHNV replication and confirming the infectivity of the stock.

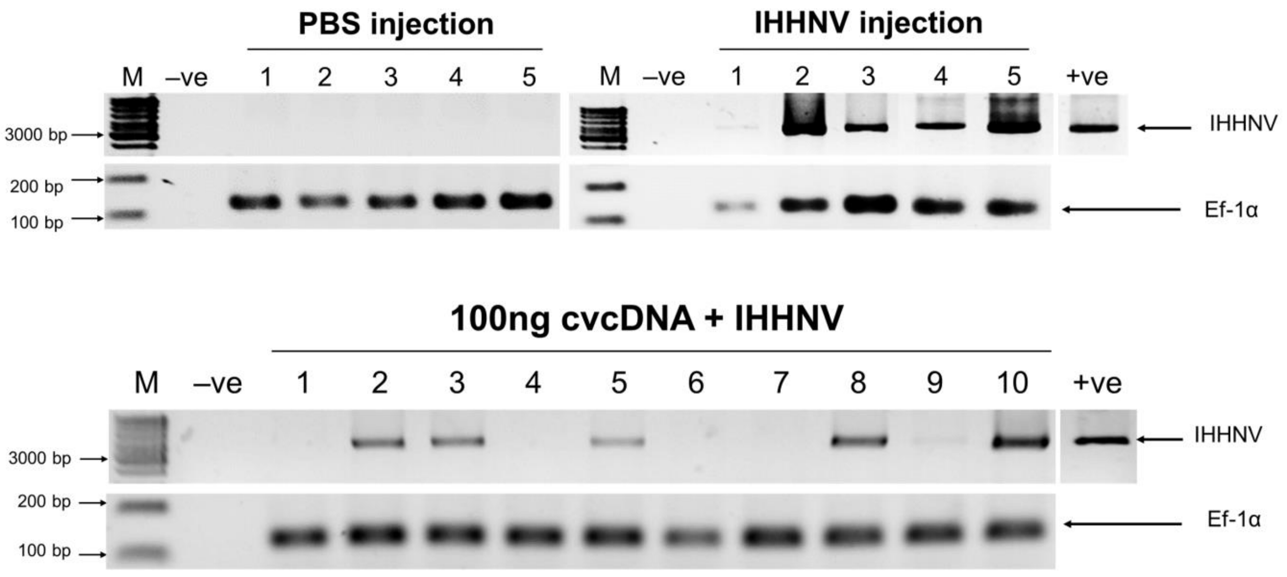

Figure S4. Photographs of inverted agarose electrophoresis gel showing IHHNV PCR amplicons from *P. vannamei* challenged with IHHNV. Long-amp PCR analysis indicating 3665 bp genomic DNA of IHHNV viral genome replication was seen only in shrimp injected with IHHNV (i.e., not the shrimp injected with PBS). Band intensities for the internal control Ef-1α were averaged and the average was used to adjust IHHNV band intensities before statistical comparison using one-way ANOVA.

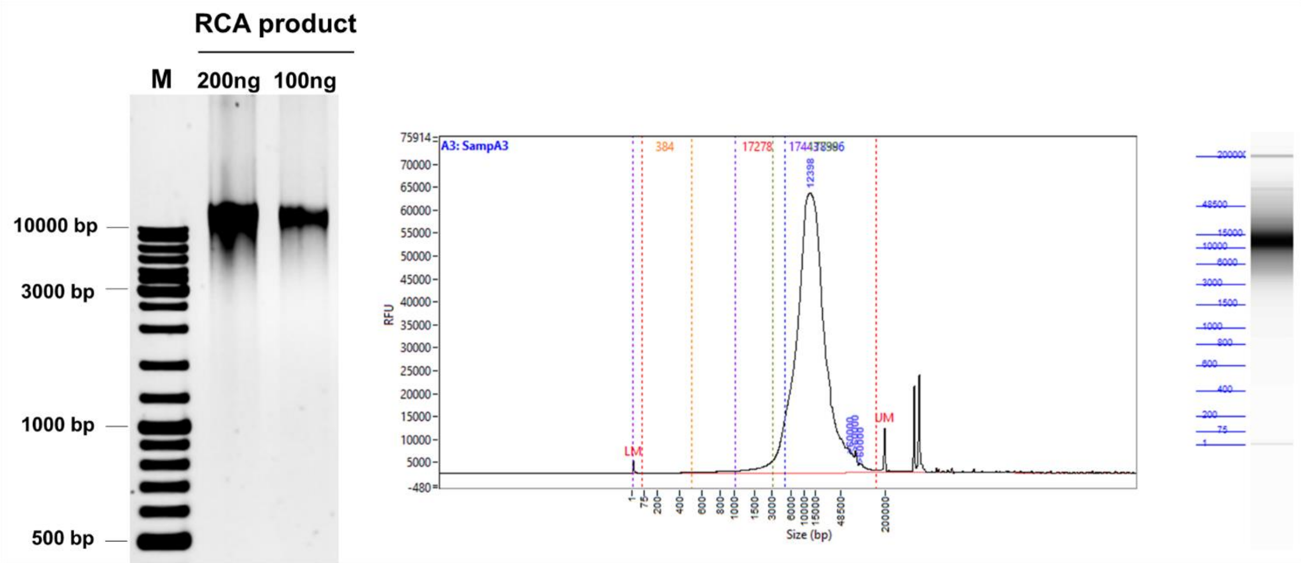

(A) **(B)**

Figure S5: Profiles of the RCA-amplified products from the cvcDNA template preparation as determined by electrophoresis. (A) Photograph of a 1.5% agarose electrophoresis gel. **(B)** Graph of the output from capillary electrophoresis. Results from both techniques revealed that the majority of the products had sizes over 11,000 bp.

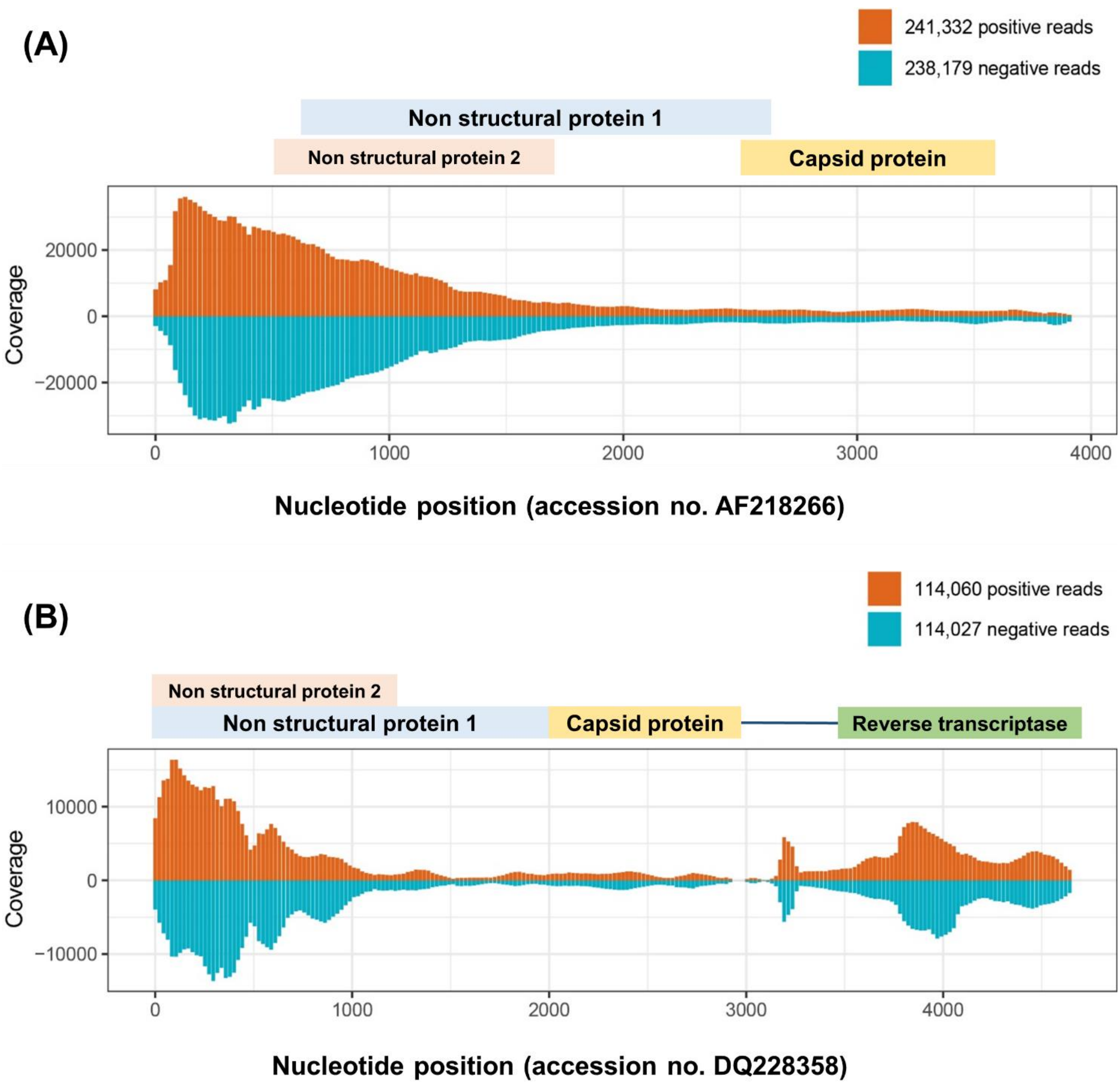

Figure S6. Diagrams of cvcDNA sequence reads distribution related to IHHNV reference sequences. The bar plots indicate distribution of the mean count of sequence reads obtained from DNA sequencing. The 21-nt sequence reads are shown as both plus and minus reads throughout the genome length. Distribution of 21-nt reads related to **(A)** GenBank accession no.AF218266 and **(B)** GenBank accession no. DQ228358. The shaded boxes above the graph represent the open reading frames of the related sequences corresponding to their nucleotide positions.

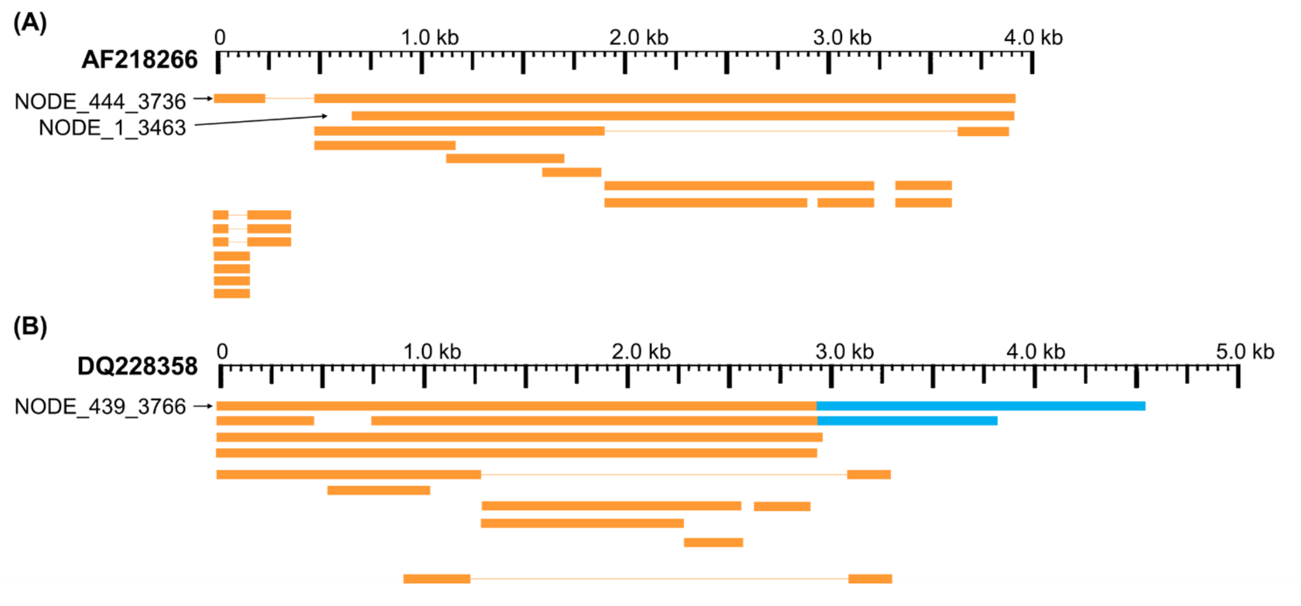

Figure S7. Schematic diagram showing the sequence similarity compared between putative cvcDNA contig sequences and GenBank records. The scale bar indicates nucleotide positions. Each box represents the sequence of an individual DNA contig. In the lowest cvcDNA in **A**, there is a long deletion (indicated by a line) when compared to the GenBank record. **(A)** cvcDNA sequences with high similarity to the IHHNV virus GenBank record AF218266. **(B)** Sequences with high similarity to non-infectious IHHNV GenBank record DQ228358. The regions in blue in (**B)** indicate the portion of the DQ228358 sequence that is part of a host shrimp transposable element and relates to the insertion point of the EVE in the shrimp genome.
